# Effects of endophytic entomopathogenic fungi on soybean aphid and identification of *Metarhizium* isolates from agricultural fields

**DOI:** 10.1371/journal.pone.0194815

**Published:** 2018-03-22

**Authors:** Eric H. Clifton, Stefan T. Jaronski, Brad S. Coates, Erin W. Hodgson, Aaron J. Gassmann

**Affiliations:** 1 Department of Entomology, Iowa State University, Ames, Iowa, United States of America; 2 United States Department of Agriculture, Northern Plains Agricultural Research Lab, Sidney, Montana, United States of America; 3 United States Department of Agriculture, Agricultural Research Service, Corn Insects and Crop Genetics Research Unit, Ames, Iowa, United States of America; Montana State University Bozeman, UNITED STATES

## Abstract

Terrestrial plants can harbor endophytic fungi that may induce changes in plant physiology that in turn affect interactions with herbivorous insects. We evaluated whether the application of entomopathogenic fungi *Beauveria bassiana* and *Metarhizium brunneum* to soybean seeds could become endophytic and affect interactions with soybean aphid (*Aphis glycines* Matsumura). It was found that *A*. *glycines* population sizes increased on plants with *M*. *brunneum* (strain F52) seed inoculum, but no significant effects were shown with analogous treatments with *B*. *bassiana* (strain GHA). Fungi recovered from soybean plant tissues indicate that endophytism was established, and that *B*. *bassiana* was more prevalent. *Metarhizium brunneum* was only recovered from stems, but *B*. *bassiana* was recovered from stems and leaves. This work confirms that some entomopathogenic fungi can be endophytic in soybean, however, some of these fungi may have a negative effect on the plants by increasing susceptibility of soybean to *A*. *glycines*. We also used DNA sequence data to identify species of *Metarhizium* obtained from agricultural fields in Iowa. Phylogenetic analyses, based on DNA sequence data, found that all isolates were *Metarhizium robertsii*, which is consistent with past studies indicating a cosmopolitan distribution and wide host range for this species. These results are important for understanding the dynamics of implementing environmentally sustainable measures for the control of pest insects.

## Introduction

Endophytic microorganisms dwell within plant tissues for at least part of their life cycle [1–3]. Some fungal endophytes may benefit plant health by enhancing growth and suppressing plant diseases and herbivorous pests, while in exchange receiving nutrition, refuge and transmission of propagules from plants [4]. Much of the early research on plant-herbivore-endophyte interactions focused on ascomycete fungi that can produce toxic alkaloids in grasses, and subsequently can cause harm to grazing livestock [5]. However, these fungal-derived alkaloids also can deter phloem-feeding insects such as the bird cherry-oat aphid, *Rhopalosiphum padi* Linnaeus, and greenbug, *Schizaphis graminum* Rondani (Hemiptera: Aphididae) [6, 7].

Another group of hypocrealean ascomycetes have long been known as arthropod pathogens. Following direct contact with potential insect hosts, entomopathogenic fungi (EPF) infect their hosts by penetrating through the cuticular exoskeleton [8]. Due to the ability to suppress pest populations and persist in the environment, EPF can serve as an alternative to chemical insecticides in certain cropping systems [9]. Some EPF strains in the genera *Beauveria* (Hypocreales: Cordyciptaceae) and *Metarhizium* (Hypocreales: Clavicipitaceae) are currently registered and marketed as biopesticides [10]. Some species of EPF used in these products are known to naturally occur in agricultural fields, including *Beauveria bassiana* and *Metarhizium robertsii*, and may sometimes cause epizootics in pest populations [11, 12].

Within cropping systems, the soil properties, plants, and potential insect hosts may affect the abundance and diversity of EPF persisting in the soil. However, identification of EPF isolates often requires molecular techniques because morphological characters can be cryptic [13–15]. To date, only a few studies on EPF diversity in cropping systems have identified isolates from soybean fields. A study in Brazil found that *Metarhizium robertsii* was the most common *Metarhizium* species in a soybean field [16]. In Maryland, USA, Kepler et al. [17] also found that *M*. *robertsii* was the most common species in a cropping system that cultivated soybean, corn, and alfalfa. By knowing what species of EPF can persist in the soils of a particular cropping system, pest management strategies can consider the potential benefits that these fungi provide in protecting crops [17].

Recent research has shown that EPF can be endophytes in a diversity of crops, including banana, maize, cotton, fava bean, poppy, tobacco, and wheat, among others [18]. Entomopathogenic fungi are typically applied to crops using foliar sprays or by soil incorporation, but endophytism can be initiated by treating the seeds or inoculating seedlings via root dips, leaf sprays, and stem injections [19–21]. Coating of maize seeds with *M*. *anisopliae sensu lato* (s.l.) conidia reduced seedling injury by wireworms and was subsequently associated with increased yields [22]. Similar studies have shown that *Metarhizium* spp. can increase root hair development in young plants, translocate nitrogen from insect cadavers, and increase the biomass of crops [23–25]. Aside from the potential benefits to plant growth, endophytic EPF may cause increased rates of infection and mortality among feeding insect pests and induce systemic plant defenses [26, 27]. In general terms, induced systemic resistance is defined as the induced state of resistance in plants, prompted by chemical or biological inducers, that primes plant defense mechanisms against future attack by pathogens or herbivorous arthropods [27]. The plant signaling molecules salicylic acid (SA), jasmonic acid (JA), and ethylene can mediate defense pathways that respond to attacks and prime the plant for future attacks [27].

Recent studies have shown that endophytic EPF may induce plant systemic resistance to insect feeding that reduces subsequent injuries caused by aphids, spider mites, and other pests [28]. The specific mechanisms underlying these systemic responses in the plant are unclear, but it has been suggested that fungal metabolites could be excreted and transported through plant vasculature, either directly affecting herbivores or mediating indirect effects through the upregulation of plant defenses [29]. Gomez-Vidal et al. [30] reported that *B*. *bassiana* endophytes could induce proteins used in plant defense and stress responses for date palm. However, the alteration of one defensive pathway in response to a pest can have an antagonistic effect on another defensive pathway, a phenomenon described as cross-talk [31]; and subsequently, can increase the susceptibility of a plant to future attacks by herbivores or pathogens.

There are some reports of endophytic EPF affecting interactions with aphids (Hemiptera: Aphididae). Inoculation of cotton, *Gossypium hirsutum* L. (Malvales: Malvaceae), with various fungi, including *B*. *bassiana* and *Lecanicillium lecanii*, significantly reduced the reproduction rate of cotton aphid, *Aphis gossypii* Glover, in greenhouse and field settings [32, 33]. An analogous study found that *B*. *bassiana* reduced populations of bean aphid, *Aphis fabae* Scopoli (Hemiptera: Aphididae) and pea aphid, *Acyrthosiphon pisum* Harris (Hemiptera: Aphididae) on fava bean, *Vicia faba* L. (Fabales: Fabaceae), but *Metarhizium anisopliae* s.l. had no effect on these aphids [34].

To date, few studies have investigated the potential effects of endophytic EPF to affect insect feeding damage on soybean, *Glycine max* L. Merr (Fabales: Fabaceae). A recent study inoculated crop plants, including soybean, with *B*. *bassiana* and recovered the fungus from leaves of plants up to 28 days after inoculation, but other tissues were not examined nor were the impacts on insect feeding included in these experiments [21]. In general, it seems that recovery of endophytic EPF decreases with time because of competition with other microorganisms and/or transience of the fungus within plant tissues [35]. In another study, soybean plants inoculated with *M*. *anisopliae* s.l. had increased the rate of plant growth under salt stress conditions compared to controls, but aspects of endophyte establishment or effects on herbivorous insects were not investigated [36].

Soybean growers in the North Central U.S. did not regularly treat their fields with insecticides until the arrival of the invasive soybean aphid, *Aphis glycines* Matsumura (Hemiptera: Aphididae), in 2000 [37]. After the establishment of *A*. *glycines*, this pest has quickly spread across most of the region, and, as a result, the amount of foliar insecticide used in soybean production has increased [38]. Alternative technologies, like resistant germplasm, are being considered for management of *A*. *glycines* [38]. Evidence suggests that soybean defenses may be suppressed by *A*. *glycines* shortly after feeding begins [39, 40], and thus, methods to enhance plant defenses may be of value in integrated pest management (IPM) for *A*. *glycines*. In the present study, we addressed the following questions: (1) Do seed treatments with either *Beauveria bassiana* or *Metarhizium brunneum* establish them as an endophyte in soybean, and for how long can these fungi be recovered from plant tissues? (2) Does EPF seed treatment, with or without endophytic establishment, affect the populations of *A*. *glycines* on soybean? (3) Which species of *Metarhizium* are present in reservoir EPF populations in agricultural fields in Iowa, including those that cultivate soybean?

## Materials and methods

### Aphid experiment

#### Experimental design

Plants used in the aphid experiment were arranged in a randomized complete block design with eight blocks, four treatments, replicated six times, for a total of 192 plants for the entire experiment. However, only five plants were used for the untreated control in one of the six replicates due to accidental damage to 11 day old plants during pot transfers, and this subsequently reduced the total sample size to 189 plants. A block was a rectangular water tray held in the growth chamber, with one plant per treatment randomly arranged on each of the eight trays (blocks). The aphid experiment was replicated six times between December 2015 and October 2016.

#### Preparation of conidial suspensions

Conidia were produced for two strains of EPF, *M*. *brunneum* F52 and *B*. *bassiana* GHA, by solid substrate fermentation as described by Jaronski and Jackson [41], and viability was determined 24 h prior to seed inoculations via germination on Sabouraud dextrose agar after 18 h of incubation at 27°C following Goettel and Inglis [42]. Conidia were suspended in autoclaved 0.10% sorbitan mono-oleate surfactant (Tween® 80) and titers were determined by a hemocytometer using diluted conidial suspensions. Soybean seeds were inoculated with suspensions of 1.0 × 10^8^ conidia mL^-1^ of *M*. *brunneum*, *B*. *bassiana*, or a 1:1 blend of *M*. *brunneum* and *B*. *bassiana*. Inoculations for the control contained the same autoclaved surfactant but no fungal conidia.

#### Inoculation of soybean seeds

Seeds of the aphid-susceptible soybean cultivar (IA3027) were obtained from the Iowa State University soybean breeding program. Seeds were briefly surface sterilized under a sterile hood in the following order: 30 s in 0.01% autoclaved Tween 80 surfactant, 60 s in 2% sodium hypochlorite with 5.5 pH, 30 s in 70% ethanol, and two consecutive rinses with sterile distilled water for 30 s each. After surface sterilization, seeds were air dried in the sterile hood on filter paper for 5 min. Seeds were placed in a sterile 50 mL conical tube (Cat. no. 14-959-49A, Fisher Scientific, Waltham, MA) containing 20 mL of a conidial suspension. Tubes containing the seeds and conidial suspensions were placed in a dark incubator at 27°C for 24 h. Tubes were laid sideways in plastic boxes in the incubator so that all seeds could be evenly distributed and soaked inside the conical tubes. If tubes sat in a vertical position, the seeds on the bottom would swell during the soaking period and push some seeds past the surface of the conidial suspension.

#### Planting and transfers

Inoculated seeds were individually planted in 266 mL plastic cups (Solo®, Dart Container Corp.,) with three small holes in the bottom for drainage. Cups were filled with unsterilized Metro-Mix® SB900 potting medium (Sun Gro Horticulture, Agawam, MA). After planting, cups with seeds received 20 mL of room temperature, deionized water, and this irrigation was repeated 4 days and 7 days after planting. Eleven days after planting, seedlings were transferred to 8 cm diameter pots containing the same SB 900 potting medium, and plants were watered as needed for the duration of the experiment. Plants were grown in a biological incubator (27°C, 60% RH, 14:10 L:D), with illumination provided by fluorescent lights (F25T8/TL841/ALTO, Philips, Amsterdam Netherlands) that produced 650 μmoles photosynthetically active radiation (PAR) m^-2^ s^-1^.

#### Inoculation with *A*. *glycines* and data collection

The *A*. *glycines* used in this study were from a biotype-1 strain initiated from individuals collected from an Iowa State University research and demonstration farm in Boone County, Iowa during July 2015. The biotype identity was confirmed using detached leaf assays [43]. *Aphis glycines* populations identified as biotype-1 are considered non-virulent and reproduce poorly on soybean cultivars with *Rag* genes (*Rag* is an abbreviation for resistance to *A*. *glycines*) compared to aphid-susceptible cultivars [44]. However, the soybean variety used in our study did not contain any *Rag* genes.

Five *A*. *glycines* nymphs of mixed age (first and second instars) were placed on each plant 14 days after seeds were planted. Nymphs were placed on the underside of the middle leaf in the first soybean trifoliate using a fine-hair paintbrush. Individual potted plants were then covered with a 53 x 51 cm, mesh net with 400 micron openings (Paint Strainer Net, Item #6LGK4, Grainger Inc., Lake Forest, IL) that was secured to the pot with a rubber band to prevent aphids from escaping. Mesh nets were carefully removed to count aphids on individual plants 1, 4, 7, 11 and 14 days after initial placement of aphids on plants (15, 19, 21, 25 and 28 days after planting).

After counting *A*. *glycines* at the last time point (day 28), the soybean biomass above the base of the stem was removed and the potting media gently shaken off the roots. Roots were then gently rinsed under a faucet. The washed roots were placed on aluminum foil trays and placed in a drying oven for 48 h at 65°C before recording the dry root mass.

### Endophyte experiment

#### Experimental design

Inoculation of IA3027 soybean seeds, planting, and incubation were performed as previously described in the aphid experiment, except that plants were not infested with aphids and were grown without the mesh coverings. Plants were arranged in a randomized complete block design with one plant per treatment randomly arranged on each of the six trays (blocks). We measured endophyte establishment at three time points (14, 21 or 28 days after planting) for the four treatments of *B*. *bassiana*, *M*. *brunneum*, a 1:1 blend of both fungi, and an untreated control. At each time point, two trays (blocks) were randomly selected and removed from the chamber to determine endophytism. The experiment was repeated four times between May 2016 and December 2016 for a total of 96 soybean plants (2 plants per treatment × 4 treatments × 3 time points × 4 replicates). Among those 96 plants, a total of 240 stem pieces and 240 leaf pieces were assessed for fungal endophytes (10 stem pieces or 10 leaf pieces per plant × 2 plants per treatment × 3 time points × 4 replicates = 240 stem pieces and 240 leaf pieces).

#### Determination of endophytism

At each time point (14, 21 or 28 days after planting), the stem and one unifoliate leaf from each plant was assessed for colonization by *B*. *bassiana* or *M*. *brunneum*. Specifically, one of the unifoliate leaves from inoculated plants and the uninoculated control plants was excised and surface sterilized in the following order with sterile 0.10% Tween 80 for 30 sec, 2% sodium hypochlorite with 5.5 pH for 2 min, 70% ethanol for 30 sec, and rinsed two times with sterile distilled water for 30 sec. Surface-sterilized leaves were first pressed onto potato dextrose agar (PDA) in a 10 cm diameter Petri dish to determine whether any conidia were present on the surface and had the potential to germinate, which could give a false positive result. If subsequent growth of the fungus was observed on the PDA agar, then any outgrowth from the corresponding plant tissues would be suspect [3, 28]. Leaves were then aseptically cut into ten 1 × 1 cm segments around the central vein of the leaf and placed onto selective media plates, with the ten leaf segments from one plant placed on the same media plate [28]. Concurrently with cutting leaves, approximately 7 to 8 cm of the soybean stem, starting from the soil surface, was cut and surface sterilized, for each plant, in the same manner as the leaves. The surface-sterilized stems were rolled on PDA plates to determine whether any conidia were present. Segments of 1 cm length were aseptically excised from both ends of the sterilized stems before aseptically cutting stems into five 1 cm pieces. Each 1 cm stem piece was cut aseptically in a longitudinal direction and each half of a 1 cm stem piece subsequently was placed onto selective media so that the inner stem surface (pith) was facing down. Similar to the media plates containing leaf segments, the ten segments of a stem from one plant were placed together on one media plate.

Selective media plates were sealed with Parafilm® and then placed in a biological incubator (27°C, 0:24 h L:D). The medium, selective for *B*. *bassiana* (GHA), was 2.0% oatmeal agar with 0.62 g L^-1^ dodine (Syllit® 65W, Platte Chemical Inc., Greenville, MS), 0.25 g L^-1^ chloramphenicol (C0378, Sigma, Saint Louis, MO), and 10 mg L^-1^ crystal violet (C6158, Sigma, Saint Louis, MO) and was based on the medium of Chase et al. [45]. To isolate *M*. *brunneum* (F52) the dodine concentration was decreased to 0.39 g L^-1^ and crystal violet was excluded. The growth of endophytic fungi from stems or leaves was determined after 11 days of incubation, and expressed as the proportion of plants with endophyte establishment, where proportion = number of plants with endophytes/total number of plants. If at least one piece of the stem or leaf had growth of endophytic fungi, that plant was recorded as positive for endophyte establishment in the stem and/or leaf.

### *Metarhizium* phylogenetics

Seventeen *Metarhizium* isolates were obtained from previous experiments described in Rudeen et al. [46] and Clifton et al. [47]. The Clifton et al. [47] study only found 12 insect cadavers with *Beauveria* infections compared to more than 190 insect cadavers with *Metarhizium* infections. As a result, the phylogenetic analysis presented here focuses on the *Metarhizium* genus which was a major cause of insect mortality in the experiments by Clifton et al. [47]. *Metarhizium* isolates came from mycosed cadavers of insects, including greater wax moth larvae, *Galleria mellonella* (Lepidoptera: Pyralidae), mealworm larvae, *Tenebrio molitor* (Coleoptera: Tenebrionidae), and western corn rootworm larvae, *Diabortica virgifera virgifera* (Coleoptera: Chrysomelidae). These insects were used in an experiment that baited entomopathogens by exposing them to soil collected from maize and soybean fields and their respective margins (S1 Table). Soil was taken from the upper 20 cm between crop rows or in the grass margins surrounding a field. More details about soil sampling and insect cadavers with *Metarhizium* spp. are described in Clifton et al. [47]. The data on mealworm cadavers was not previously published in the study by Clifton et al. [47] because mealworms were only used in one of the two years of that study. Insect cadavers with *Metarhizium* conidia were kept in 1.5 mL microfuge tubes at -20°C after the experiments were performed in 2011 and 2012. In 2016, *Metarhizium* isolates were cultured from insect cadavers by transferring the fungus to agar medium following Goettel and Inglis [42]. A small surface area containing conidia on the insect cuticle, approximately 1 mm^2^, was swabbed with a sterile cotton-tip applicator and transferred to Petri dishes containing Sabouraud dextrose agar (SDA). After 14 days of growth the *Metarhizium* isolates were stored at 4°C until further use.

DNA extractions were performed using a cetyltrimethyl ammonium bromide (CTAB) protocol with slight modifications [48]. For this 7 day old cultures of each *Metarhizium* isolate were prepared so that a dense mat of mycelium had developed on the surface of a Petri dish containing oatmeal dodine agar. Mycelia were scraped from the surface of the medium, flash frozen with liquid nitrogen, and ground in CTAB extraction buffer. This mixture was transferred to sterile 1.5 mL microfuge tubes and incubated overnight on a heating rack set at 55°C with 50 ng ul^-1^ proteinase K. Extracts were further purified by the addition of 200 μL 1:1 phenol:chloroform, vortexed vigorously, centrifuged at 14,000 rpm for 5 min, and then the top aqueous phase carefully transferred to a new 1.5 mL tube. DNA was precipitated from solution by the addition of an equal volume of isopropanol and a sodium chloride solution at a final concentration of 0.2 M, after which it was vortexed, and then centrifuged at 14,000 rpm for 20 min in a 4°C refrigerated centrifuge (model 5417R, Eppendorf, Hamburg, Germany). The DNA pellets were washed with 70% ethanol, dried, suspended in 150 μL distilled deionized water, and stored at -20°C.

Polymerase chain reaction (PCR) assays were carried out using primers for elongation factor 1-alpha (EF-1α) and β–tubulin. To identify species of *Metarhizium*, we compared our isolates to a subset of *Metarhizium* isolates described in Bischoff et al. [14] that have sequence data for the same genes on GenBank (S2 Table). The primers used to amplify these genes were the same as those used in Bischoff et al. [14]. Amplification of EF-1α was performed in 40 μL reaction volumes consisting of 1X GoTaq® Flexi Buffer (Promega, Madison, WI), 1 μM of each primer, 3 mM MgCl_2_, 200 μM dNTP, 2U *Taq* DNA polymerase, and 20 ng genomic DNA. Thermocycler conditions consisted of a touch-down protocol with 30 s initial denaturation at 94°C followed by 10 cycles of 10 s at 94°C, 30 s at 65–55°C (reducing annealing temperature by 1°C per cycle), and 30 s at 72°C. Subsequently, 35 cycles were performed with the same conditions, however, with a fixed annealing temperature of 55°C, a final extension of 10 min at 72°C, followed by a 4°C hold until PCR products were used in gel electrophoresis or stored at -20°C. Amplification of β-tubulin was performed with the same reaction volumes and reagents, but thermocycler conditions consisted of 40 cycles of 35 s at 94°C, 55 s at 52°C, and 2 min at 72°C, followed by a 4°C hold. Electrophoresis of 5 μL for each PCR product was performed on 1.5% agarose gel containing 0.70 mg ethidium bromide L^-1^, and products were visualized with a UV transilluminator (model M-20E, UVP, Upland, CA). The remaining 35 μL of successful PCR products were purified using the QIAquick PCR purification kit (Cat. No. 28104, Qiagen, Germany), and submitted to the DNA Facility at Iowa State University for Sanger DNA sequencing with an Applied Biosystems 3730 DNA analyzer (Life Technologies Corporation, Carlsbad, CA). Sequence data were individually analyzed in SeqTrace 0.9.0 software [49] to confirm sequence quality and to trim ambiguous ends that lacked consensus.

Phylogenetic analyses included EF-1α and β-tubulin sequence data from a total of 45 *Metarhizium* isolates. Seventeen of these isolates were from samples collected in Iowa (S1 and S2 Tables) and the remaining 28 isolates were described in Bischoff et al. [14] and were downloaded from GenBank [50]. The isolates from Bischoff et al. [14] consisted of DNA sequence for eight species of *Metarhizium*: *M*. *acridum*, *M*. *anisopliae*, *M*. *brunneum*, *M*. *guizhouense*, *M*. *lepidiotae*, *M*. *majus*, *M*. *pingshaense*, and *M*. *robertsii*. The DNA sequence from individual genes were concatenated by isolate, and a multiple sequence alignment generated with MEGA 6.0 [51] using the Clustal W algorithm with the default settings. The best model tool in MEGA 6.0 was used to select the Jones-Taylor-Thornton (JTT) model for sequence evolution, and the subsequent phylogeny was inferred using the maximum likelihood method with 1000 bootstrap pseudoreplicates [52], with all aligned positions containing gaps and missing data omitted.

### Data analysis

Data on the number of aphids per plant for each data collection period were converted to cumulative aphid days (CAD) per plant following Ruppel [53] and Hodgson et al. [54], which provides a single value for the total aphid abundance on a plant over the duration of the experiment. Data on CAD and dry root mass were analyzed with SAS Enterprise Guide 6.1 software [55]. Cumulative aphid days were transformed with the log_10_ function to normalize residuals. Data were analyzed with a mixed-model of analysis of variance (ANOVA) (PROC MIXED). The model used the fixed effect of treatment (control, *B*. *bassiana*, *M*. *brunneum*, or *B*. *bassiana*: *M*. *brunneum* blend). The random effects were the experimental run and the interaction of run with the fixed effect of treatment. When the effect of treatment was significant, pairwise comparisons were made using the PDIFF statement in PROC MIXED. Pairwise comparisons were based on least-square means with an experimentwise error rate of *P* < 0.05 after using the Bonferroni adjustment for six comparisons.

For the endophyte experiment, the frequency of plants with successful recovery of endophytic EPF was analyzed with a G-test of independence using the PROC FREQ statement in SAS 9.4 [56]. The proportion of plants with or without endophytes were combined for all time points and were analyzed separately by soybean tissue (stem vs. leaf). We combined data from the single treatments and blend treatment and then compared the frequency of *B*. *bassiana* endophytes to *M*. *brunneum* endophytes. We also compared the frequency of *B*. *bassiana* endophytes in the single treatment to the frequency of *B*. *bassiana* endophytes in the 1:1 blend treatment to determine if a combination of inoculum affected endophyte recovery. The same comparison between the single treatment and 1:1 blend treatment was performed for *M*. *brunneum* endophytes. Finally, we used the PROC FREQ statement to compare the proportion of plants with *B*. *bassiana* endophytes at different time points.

## Results

**Fig 1:**
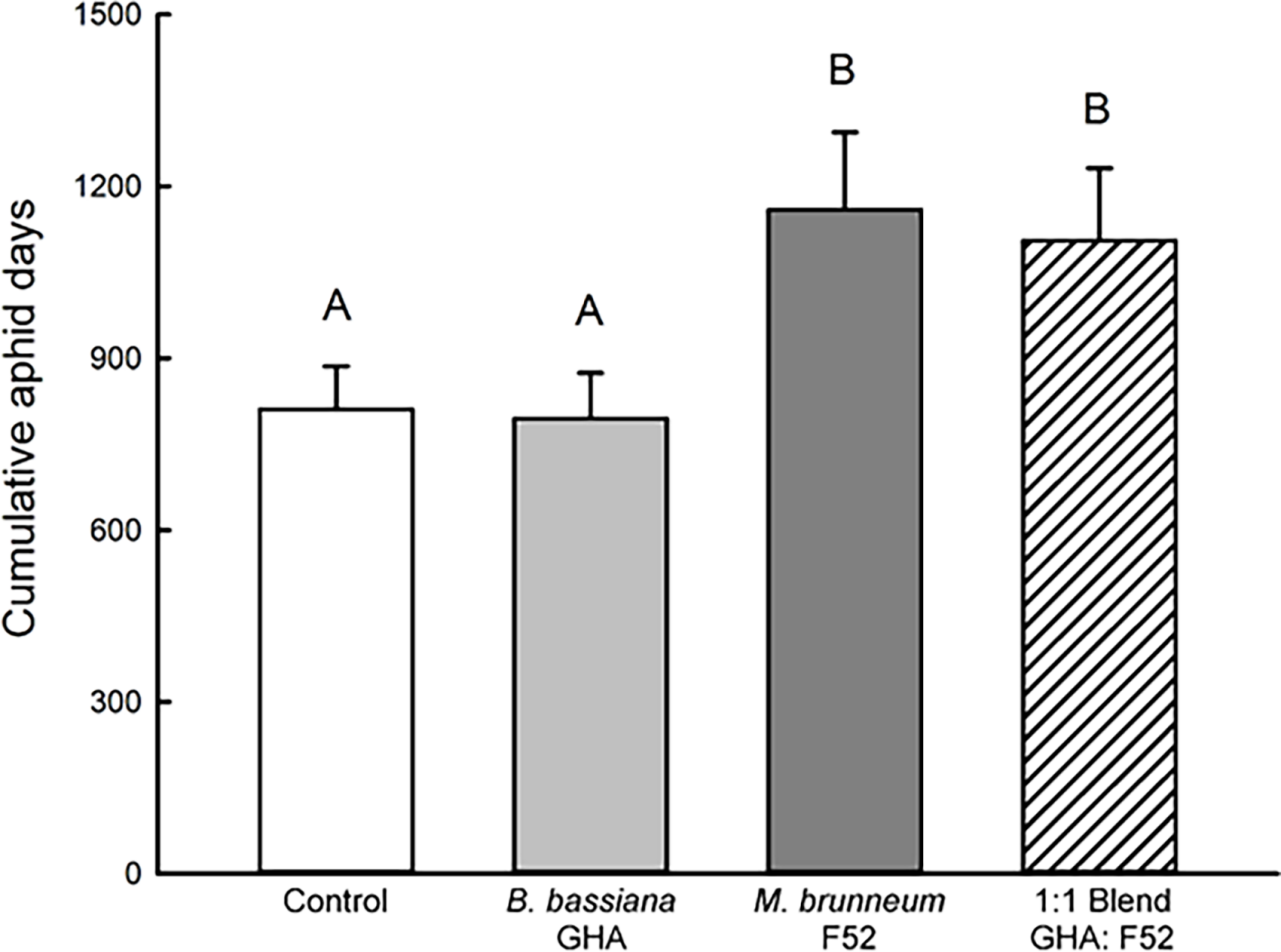
Cumulative aphid days for soybean aphids (*Aphis glycines*) on soybean plants. Bar heights are sample means and error bars are the standard error of the mean. Letters above the bars represent significant differences between means.

For CAD, there was a significant effect of treatment on *A*. *glycines* populations (df = 3, 15; F = 8.68; *P* = 0.0014; Fig 1). The treatment with *B*. *bassiana* GHA alone did not differ from the untreated control (df = 15; t = 0.48; *P* = 1.00). However, both the treatment with *M*. *brunneum* F52 alone and the 1:1 blend had significantly greater CAD than the untreated control and the treatment with *B*. *bassiana* GHA alone (Fig 1). We did not find a significant effect of treatment on dry root mass (df = 3, 15; F = 1.11; *P* = 0.3819; S1 Fig).

**Fig 2:**
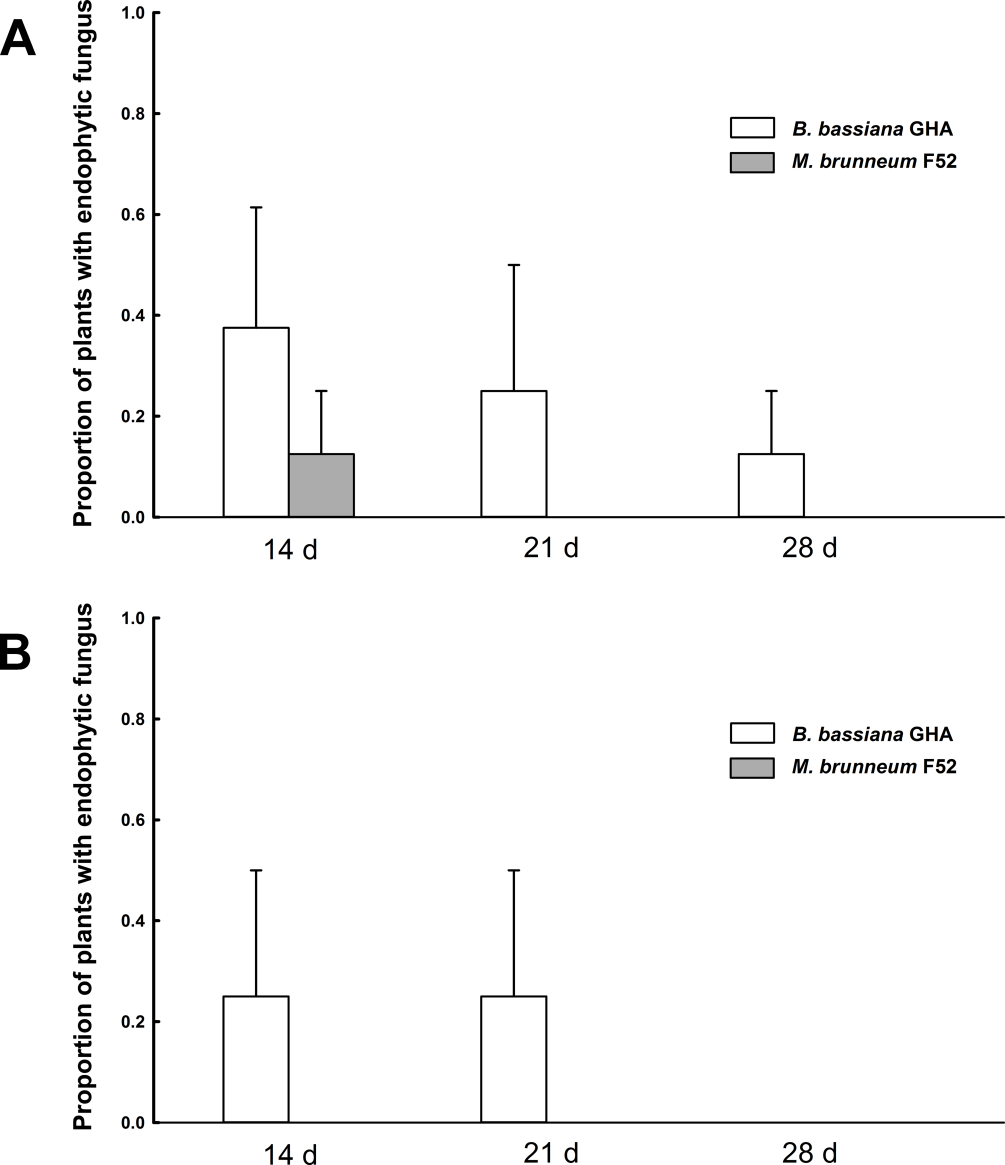
Endophyte recovery in soybeans inoculated with a single inoculum. Mean proportion of plants with endophyte recovery in (A) soybean stems and (B) soybean leaves for plants inoculated with either *Beauveria bassiana* GHA alone or *Metarhizium brunneum* F52 alone. Bar heights represent the sample mean and error bars are the standard error of the means. Bars are separated by the time points of 14, 21 or 28 days after planting. The shading in bars represent the species of fungus recovered.

**Fig 3:**
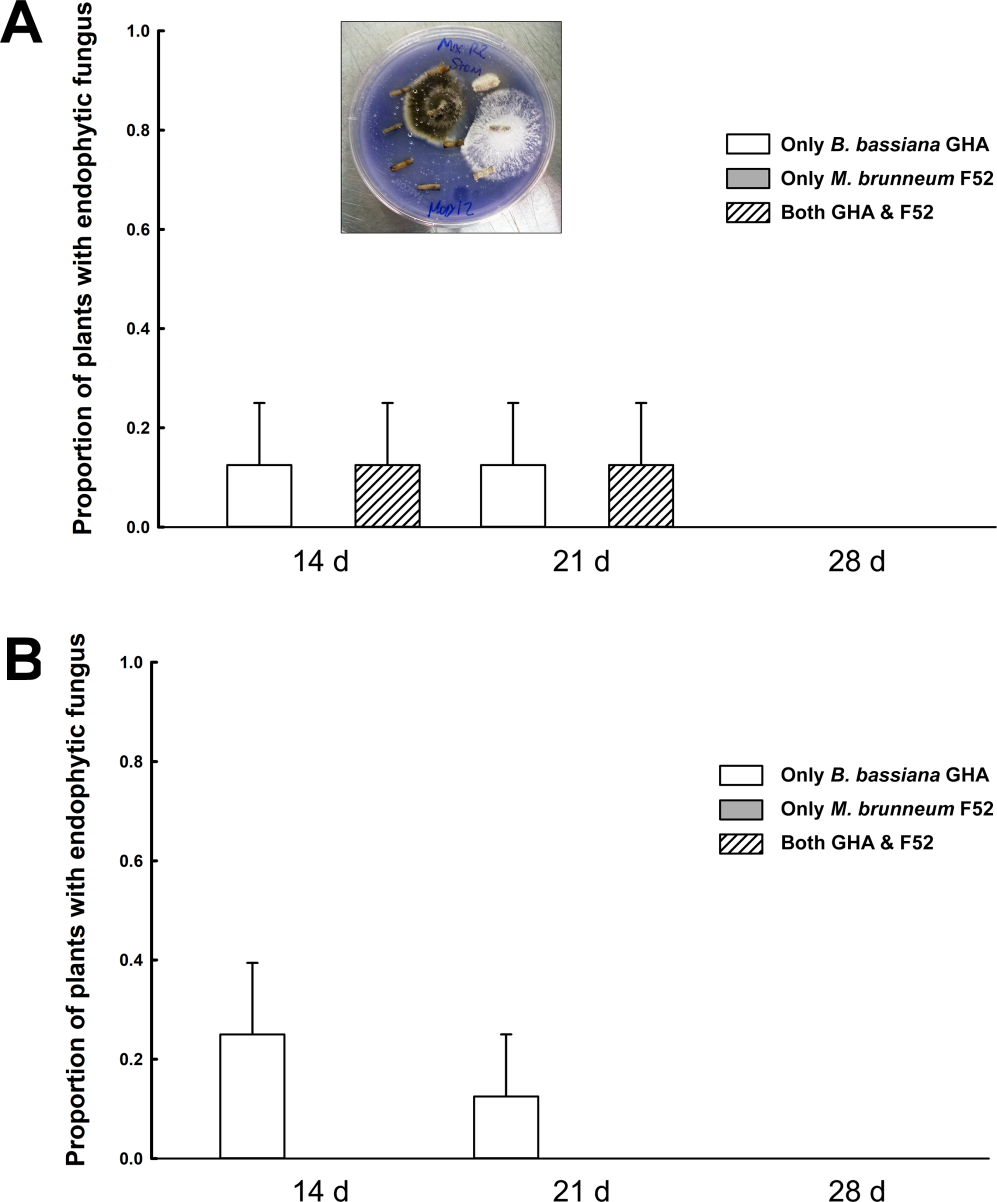
Endophyte recovery in soybeans inoculated with the blend inoculum. Mean proportion of plants with endophyte recovery in (A) soybean stems and (B) soybean leaves in the 1:1 blend treatment of *Beauveria bassiana* GHA and *Metarhizium brunneum* F52. Bar heights represent the sample mean and error bars represent are the standard error of the means. Bars are separated by the time points of 14, 21 or 28 days after planting. The shading in bars represents the species of fungus that was recovered. Image is included showing endophytic *B*. *bassiana* and *M*. *brunneum* being recovered from separate stem pieces in the same soybean plant.

All of the PDA plates that received leaf presses or stem rolls from surface-sterilized plants had no growth of *B*. *bassiana* or *M*. *brunneum*, suggesting that no viable conidia were present on plant surfaces following sterilization to give a false positive result for detection of fungal endophytes. Overall, the occurrence of *B*. *bassiana* as an endophyte was significantly greater than *M*. *brunneum* (df = 1, *G* = 4.57, *P* = 0.0325). The percentage values that we report for plants with endophytes in the stems or leaves are equivalent to the values for proportion (shown in Figs 2 and 3) multiplied by 100. In total, with all time points combined, *B*. *bassiana* was recovered from the stems in 10 out of 48 inoculated plants (20.8%), while *M*. *brunneum* was only recovered from the stems in 3 out of 48 inoculated plants (6.25%) (Figs 2A and 3A). For leaf tissue, we recovered *B*. *bassiana* in 7 out of 48 inoculated plants (14.58%), while *M*. *brunneum* was recovered from zero out of 48 inoculated plants (Figs 2B and 3B). Among the seven plants that had *B*. *bassiana* endophytes in their leaves, six of them also had *B*. *bassiana* endophytes in the stems. Another result worth noting is that *M*. *brunneum* was not recovered alone as an endophyte in the stems of plants grown from the 1:1 blend treatment, but rather *M*. *brunneum* was recovered from plants that also had *B*. *bassiana* endophytes in the stems. The frequency of *B*. *bassiana* recovery, from the single inoculum treatment, did not differ significantly from its frequency as an endophyte in the 1:1 blend treatment (df = 1, *G* = 0.51, *P* = 0.48), and the same pattern was observed for *M*. *brunneum* (df = 1, *G* = 0.36, *P* = 0.55). The frequency of *B*. *bassiana* recovery, from the single inoculum treatment, did not differ significantly among time points in the stems (df = 2, *G* = 1.38, *P* = 0.50), or in the leaves (df = 2, *G* = 3.63, *P* = 0.16). With the exception of a few plants treated with *B*. *bassiana*, the levels of endophyte recovery by day 28 were zero in stems and leaves. It is also worth noting that we did not recover endophytes from any plants in one of the four experimental runs.

**Fig 4:**
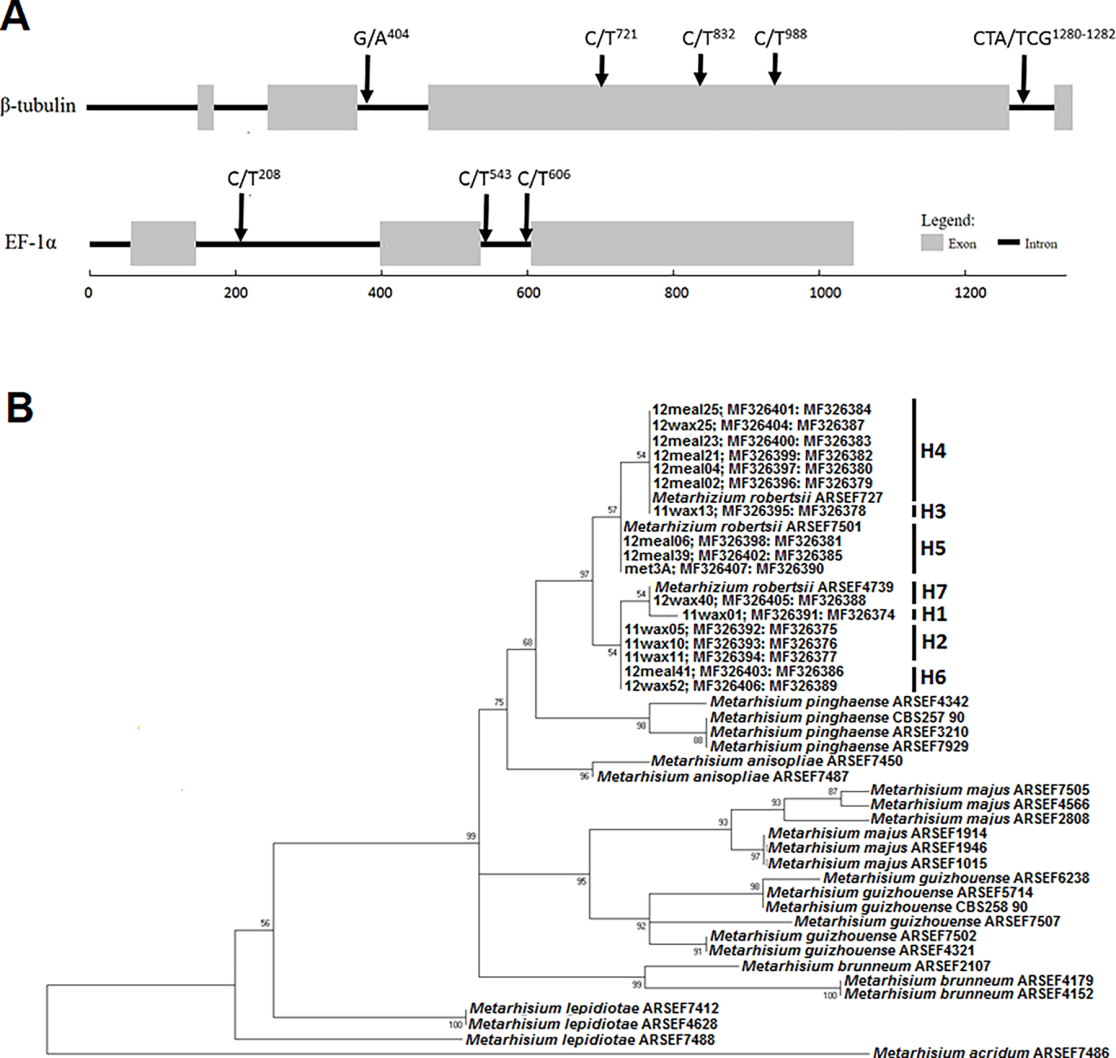
Phylogenetic analysis of *Metarhizium* isolates. **A)** Schematic of PCR amplified beta-tubulin (β-tubulin) and elongation factor alpha 1-alpha (EF-1α) showing positions of substitution mutations among isolates in this study. **B)** Maximum-likelihood estimated phylogenetic relationships among *Metarhizium* isolates based on analysis of aligned concatenated β-tubulin and EF-1α sequences Branch points for isolates in this study are listed with corresponding GenBank accession numbers (isolate; β-tubulin: EF-1α), where four unique haplotypes (H1 to H7) are indicated. Isolate collection information is provided in S1 Table. Bootstrap values (1,000 replicates) are indicated beside the nodes. The tree is rooted to *Metarhizium acridum*. The tree is drawn to scale, with branch lengths measured in the number of substitutions per site.

The aligned DNA sequence data identified seven unique haplotypes among the 17 isolates, of which five were located in the β-tubulin gene and three transition mutations were observed in the EF-1α gene (Fig 4A; S2 Fig). Three mutation sites in the β-tubulin gene were located in an exon, whereas the other substitution sites, and all substitution sites in EF-1α occurred in the introns. The mutation sites were all synonymous substitutions that were not predicted to change the final amino acid sequences. Subsequent phylogenetic analysis separated *Metarhizium* species into different clades with strong bootstrap support, and all of the *Metarhizium* field isolates from this study clustered within a single clade along with *Metarhizium robertsii* (Fig 4B).

## Discussion

Goals of this study were to determine whether EPF can establish an endophytic relationship with soybean and if inoculation of seeds with EPF impacts the pest *A*. *glycines*. It was found that plants inoculated with *M*. *brunneum* alone or in the blend treatment (1:1 mixture of *M*. *brunneum* and *B*. *bassiana*) had overall greater abundance of *A*. *glycines* compared to the plants inoculated with *B*. *bassiana* or the untreated control (Fig 1). A significantly lower proportion of plants were endophytic with *M*. *brunneum* as compared to *B*. *bassiana* (Figs 2 and 3). Additionally, *Metarhizium brunneum* was only isolated from stems whereas *B*. *bassiana* was isolated from stems and leaves, and both fungi were recovered from the stems of individual plants treated with the 1:1 blend (Fig 3). The observation of transiency, as well as the overall low levels of positive endophytism, matches the results of Russo et al. [21] where the observed levels of *B*. *bassiana* endophytes from a seed inoculation started at low levels and gradually decreased over 28 days in soybean plants. The additional goal of this study aimed to identify the species of soil-dwelling entomopathogenic *Metarhizium* within maize and soybean cropping systems, for which all of those isolated were *M*. *robertsii* (Fig 4).

Two potential explanations may be hypothesized for the increased aphid abundance on plants inoculated with *M*. *brunneum*: 1) *M*. *brunneum* could potentially reduce the efficacy of general plant defenses, or 2) that *M*. *brunneum* could improve host-plant quality for aphids. It is possible that soybean plants may have responded to endophytic *M*. *brunneum* via up-regulation of a pathogen defensive pathway, potentially mediated by salicylic acid (SA), and the subsequent cross-talk between the SA pathway and the jasmonic acid (JA) pathway [31, 57]. This plant response could have rendered plants less capable of defending against the additional stress of aphid attack. The observed lower levels of endophytism by *M*. *brunneum* compared to *B*. *bassiana* suggests that soybean plants did not support the presence of *Metarhizium* endophytes the same as *Beauveria*, and that plants may have removed *M*. *brunneum* with mechanisms that also are employed to remove saprophytic pathogens, many of which are regulated by the SA pathway [27, 58]. Quesada-Moraga et al. [59] suggests that *B*. *bassiana* can easily ascend from the rhizosphere to distal aboveground tissues after inoculated plants have had the time to mature. It was previously shown that endophytic *Metarhizium* spp. can increase root hair density and enhance nutrient acquisition by plants [23–25], and it may be possible that *M*. *brunneum* elicited analogous responses among soybean plants in this experiment, although no differences in root biomass were observed among endophyte treated and control plants (S1 Fig).

Plants are not passive bystanders to pathogen attacks, but are capable of recognizing microbial compounds (i.e., fungal chitin) that trigger defensive pathways [60, 61]. Beneficial fungi and saprophytic pathogens have evolved adaptive mechanisms, such as the secretion of effector proteins, to facilitate the evasion of plant defenses and allow their colonization of extracellular spaces within plant tissues [62]. Schulz and Boyle [3] described this phenomenon as “balanced antagonism” to explain the interaction between host plant and endophyte. In this study, it was observed that *M*. *brunneum* elicited a soybean plant response that facilitated a significantly greater abundance of *A*. *glycines*. Past research has suggested that EPF, particularly *B*. *bassiana*, may induce plant defenses that in turn suppress insects [28, 33], however analogous results were not observed for *A*. *glycines* in this study. Akello and Sikora [34] used a method, similar to ours, of seed-soaking fava bean and found that *M*. *anisopliae* s.l. had no effect on *A*. *fabae* and *A*. *pisum* aphids, whereas *B*. *bassiana* significantly reduced aphid fecundity. The high concentration of *M*. *brunneum* conidia in the initial seed-soak inoculum may have resulted in upregulated SA-mediated defenses during later plant development which are often triggered in response to saprophytic pathogens, e.g. *Fusarium* spp. or *Pythium* spp. [63]. It may thus be hypothesized that the ensuing upregulation of the SA and/or JA pathways could potentially have resulted in a plant physiological state that was more susceptible to insect feeding, potentially due to a period of cross-talk between SA and JA pathways. In a period of cross-talk, plants infected by SA-inducing pathogens, or in this case plants inoculated with EPF, could suppress JA-dependent defenses used for insect herbivores [31]. Unlike the leaf-chewing insects that may increase levels of endogenous JA in plants, *A*. *glycines* can trigger SA-marker genes when feeding on susceptible soybeans [64], so it is unclear how the plants in this study responded to aphids after a period of growth and inoculation with fungi. Furthermore, *A*. *glycines* is capable of hijacking plant defense pathways in soybean [39] and inducing a state of susceptibility in resistant cultivars that allows for greater *A*. *glycines* reproduction [40]. However, the levels of endogenous SA or JA were not measured in soybean tissues for this study, and the hypothesis of alterations to plant defenses [65] remains to be tested as an explanation of the data in the current study.

It is also possible that *M*. *brunneum* may have increased nutrient levels in soybeans. Previous studies have observed that *B*. *bassiana* and *M*. *anisopliae* s.l. can colonize a wide range of crops, including leguminous plants, and promote plant growth [36, 66–69]. In general, *B*. *bassiana* seems capable of establishing as an endophyte aboveground in the phylloplanes of many plants, whereas *Metarhizium* spp. tend to establish as endophytes in roots [12, 70, 71]. As we saw in our study, *B*. *bassiana* was able to establish in soybean stems and sometimes make its way into the leaf tissue of the same plants. In a study of EPF inoculations on soybean, Khan et al. [36] showed that *M*. *anisopliae* s.l. improved soybean shoot length and shoot dry weight when plants were grown under salt-stressed conditions. Endophytic EPF may increase plant growth in a way that allows plants to better tolerate insect herbivory and compensate for lost biomass [28, 72]. Behie et al. [23] showed that *M*. *robertsii* could translocate nitrogen from soil-borne insect cadavers to haricot bean seedlings. Additionally, aphids have been observed to be more fecund and increase their populations more rapidly on plants supplemented with nitrogen fertilizer [73]. Thus, it may be the case that *M*. *brunneum* increased nutritional quality of soybean plants for aphids and future studies could explore the effects of this fungus on soybean development. However, it is unknown to what extent *M*. *brunneum* may have altered host-plant quality in this experiment because there were no significant effects on root biomass and other plant growth parameters were not measured (S1 Fig).

The natural environment harbors a large diversity of EPF that may affect interactions between arthropods and plans, and understanding the diversity and persistence of EPF within a particular agroecosystem is important in determining the relevant strains for further study. Our phylogenetic analysis placed all our *Metarhizium* isolates within clades with *M*. *robertsii* (Fig 4B). Rehner and Kepler [15] recently described the phylogeography of the *M*. *anisopliae* complex and found that *M*. *robertsii* is most predominant in North America as compared to other global regions, and this distribution could reflect historical differences in species origin or ecological adaptations. Previous studies have shown that different species of *Metarhizium* may not be randomly distributed in soil environments, but rather exhibit a degree of specificity for the rhizosphere of certain plants [74], but also vary by climate, soil type, and cultivation practices [75, 76]. Kepler et al. [17] found higher numbers of *Metarhizium* colony forming units in field plots with soybean cultivation compared to corn or alfalfa, and a majority of the isolates used in their phylogenetic study were identified as *M*. *robertsii*. Behie et al. [66] obtained hundreds of *Metarhizium* isolates from the roots of grasses, forbs and sedges in Canada, and identified 95% of the *Metarhizium* isolates as *M*. *robertsii*. The original collection of isolates used in the current study [47] were not sampled from soil within the rhizosphere of maize or soybean roots, but we obtained isolates from cadavers infected by EPF within bulk soil that was collected adjacent to crop rows. Regardless, all of these isolates were identified as *M*. *robertsii* (Fig 4B), suggesting this species may be the most effective at persisting in North American field environments. Despite the apparent prevalence of *M*. *robertsii* compared with other *Metarhizium* spp. in North America, soil sampling methods and the insects used to bait entomopathogens could bias the *Metarhizium* isolates that were obtained [15–17].

*Metarhizium robertsii* is known to infect a variety of insect hosts and in different insect orders [15], and our isolates infected hosts belonging to both Coleoptera and Lepidoptera (S2 Fig). Other studies on EPF isolates and haplotypes observed distinct groups based on host insect range [77–79]. Hernández-Domínguez and Guzmán-Franco [80] collected *Metarhizium* isolates from one sugarcane field and found that the frequency of specific haplotypes had significantly varied by the date of sampling, suggesting that temporal variation also may need to be considered when devising a collection strategy.

In terms of management of *A*. *glycines*, it remains unclear whether microorganisms like EPF could help to protect soybeans from aphids. Lopez et al. [33] found that *B*. *bassiana* were able to suppress *A*. *gossypii* populations on cotton in both laboratory and field settings, suggesting that some fungal endophytes could be useful tools in management of agricultural pest insects. A recent review by McKinnon et al. [28] suggested that *B*. *bassiana* endophytes tend to have positive impacts on plant growth and negative impacts on insect pests, but a similar review on *Metarhizium* is lacking. Regardless of trends observed in these studies, the tritrophic interactions among different host plants, insects and fungal entomopathogens can be very specific and complex. *Metarhizium brunneum* F52 was used in these experiments but we did not identify any of our field-collected isolates as *M*. *brunneum*. Other species of *Metarhizium* also might establish as soybean endophytes and their potential impacts on soybeans and *A*. *glycines* could be different. Because this study determined that *M*. *robertsii* can be found in soybean fields and because of its wide distribution in North America [15–17, 66], future studies should consider the ecological roles of *M*. *robertsii* in row crop agriculture, including its effects on arthropod-plant interactions and its application as a possible biocontrol agent within IPM programs. Future studies also should determine the potential impacts of fungal endophytes on crops with host-plant resistance genes, which are already considered as a key tool in IPM [81], especially for pests like *A*. *glycines* [37].

## Supporting information

S1 Fig Dry root mass of soybean plants. Bar heights are sample means and error bars are the standard error of the mean.
(TIF)

S2 Fig Transition sites in *Metarhizium* isolate genes. Sites of transition mutations in the genes amplified for our *Metarhizium* isolates that made up the seven haplotypes.
(TIF)

S1 Table *Metarhizium* isolates from Iowa cropping systems. *Metarhizium* isolates used in the phylogeny study, their location in Iowa, their host before media culture, and the previous study from which they came. Elongation factor 1-alpha (EF-1α) and β–tubulin (Bt) genes were sequenced for all isolates.
(DOCX)

S2 Table Reference *Metarhizium* isolates. Reference sequences used for phylogenetic analyses; includes strain codes, taxon name, host, country of collection, and GenBank accession numbers for the subset of isolates picked from Bischoff et al. (2009).
(DOCX)
