# Supplementary figures and images for: Effects of endophytic entomopathogenic fungi on soybean aphid and identification of Metarhizium isolates from agricultural fields
Source: PLoS One. 2018 Mar 22;13(3):e0194815. doi: 10.1371/journal.pone.0194815 (PMC5864058; doi:10.1371/journal.pone.0194815)

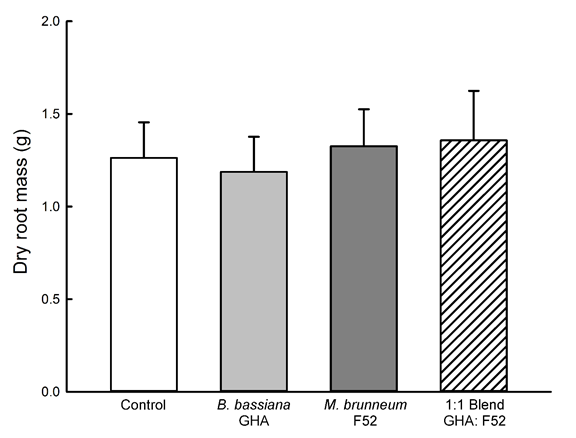

Supplement: S1 Fig — Bar heights are sample means and error bars are the standard error of the mean. (TIF) [file pone.0194815.s001.tif]

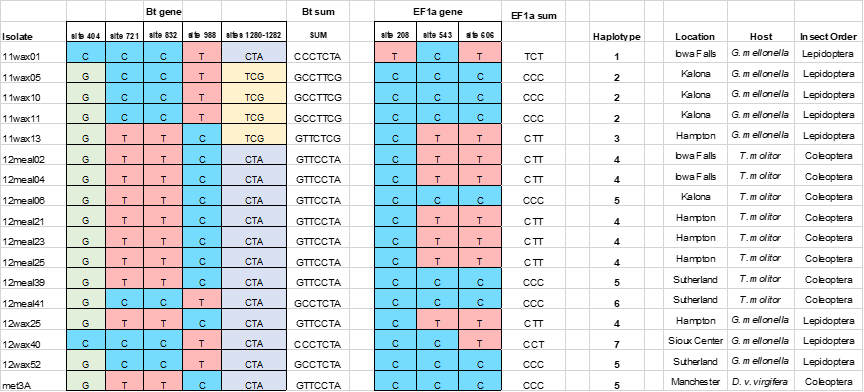

Supplement: S2 Fig — Sites of transition mutations in the genes amplified for our Metarhizium isolates that made up the seven haplotypes. (TIF) [file pone.0194815.s002.tif]
