# Supplementary material for: Effects of endophytic entomopathogenic fungi on soybean aphid and identification of Metarhizium isolates from agricultural fields
Source: PLoS One. 2018 Mar 22;13(3):e0194815. doi: 10.1371/journal.pone.0194815 (PMC5864058; doi:10.1371/journal.pone.0194815)
Supplement: S1 Table — Metarhizium isolates used in the phylogeny study, their location in Iowa, their host before media culture, and the previous study from which they came. Elongation factor 1-alpha (EF-1α) and β–tubulin (Bt) genes were sequenced for all isolates. (DOCX) [file pone.0194815.s003.docx]

**Supplementary Table 1**: *Metarhizium* isolates used in the phylogeny study, their location in Iowa, their host before media culture, and the previous study from which they came. Elongation factor 1-alpha (EF-1α) and β–tubulin (Bt) genes were sequenced for all isolates.

| **Isolate ID** | **Cropping system and location** | **Nearby city** | **Isolation source** | **Order** | **Study** | **Bt^a^** | **EF-1α^b^** |
| --- | --- | --- | --- | --- | --- | --- | --- |
| 11wax01 | Conv^a^ corn field | Iowa Falls | *G. mellonella* | Lepidoptera | Clifton et al. (2015) | MF326391 | MF326374 |
| 11wax05 | Organic corn margin | Kalona | *G. mellonella* | Lepidoptera | Clifton et al. (2015) | MF326392 | MF326375 |
| 11wax10 | Organic soybean field | Kalona | *G. mellonella* | Lepidoptera | Clifton et al. (2015) | MF326393 | MF326376 |
| 11wax11 | Organic soybean margin | Kalona | *G. mellonella* | Lepidoptera | Clifton et al. (2015) | MF326394 | MF326377 |
| 11wax13 | Organic soybean field | Hampton | *G. mellonella* | Lepidoptera | Clifton et al. (2015) | MF326395 | MF326378 |
| 12meal02 | Conv soybean field | Iowa Falls | *T. molitor* | Coleoptera | Clifton et al. (2015) | MF326396 | MF326379 |
| 12meal04 | Conv soybean margin | Iowa Falls | *T. molitor* | Coleoptera | Clifton et al. (2015) | MF326397 | MF326380 |
| 12meal06 | Conv corn field | Kalona | *T. molitor* | Coleoptera | Clifton et al. (2015) | MF326398 | MF326381 |
| 12meal21 | Conv soybean field | Hampton | *T. molitor* | Coleoptera | Clifton et al. (2015) | MF326399 | MF326382 |
| 12meal23 | Conv soybean field | Hampton | *T. molitor* | Coleoptera | Clifton et al. (2015) | MF326400 | MF326383 |
| 12meal25 | Conv soybean field | Hampton | *T. molitor* | Coleoptera | Clifton et al. (2015) | MF326401 | MF326384 |
| 12meal39 | Conv soybean field | Sutherland | *T. molitor* | Coleoptera | Clifton et al. (2015) | MF326402 | MF326385 |
| 12meal41 | Conv soybean margin | Sutherland | *T. molitor* | Coleoptera | Clifton et al. (2015) | MF326403 | MF326386 |
| 12wax25 | Conv soybean field | Hampton | *G. mellonella* | Lepidoptera | Clifton et al. (2015) | MF326404 | MF326387 |
| 12wax40 | Organic soybean margin | Sioux Center | *G. mellonella* | Lepidoptera | Clifton et al. (2015) | MF326405 | MF326388 |
| 12wax52 | Conv soybean margin | Sutherland | *G. mellonella* | Lepidoptera | Clifton et al. (2015) | MF326406 | MF326389 |
| Met3A | Conv corn field | Manchester | *D. v. virgifera* | Coleoptera | Rudeen et al. (2013) | MF326407 | MF326390 |
| ^a^ Bt: GenBank accession number for β-tubulin sequence data  ^b^ EF-1α: GenBank accession number for elongation factor 1-alpha sequence data  ^c^ Conv: conventional farming production | | | | | | | |
