# Supplementary material for: Effects of endophytic entomopathogenic fungi on soybean aphid and identification of Metarhizium isolates from agricultural fields
Source: PLoS One. 2018 Mar 22;13(3):e0194815. doi: 10.1371/journal.pone.0194815 (PMC5864058; doi:10.1371/journal.pone.0194815)
Supplement: S2 Table — Reference sequences used for phylogenetic analyses; includes strain codes, taxon name, host, country of collection, and GenBank accession numbers for the subset of isolates picked from Bischoff et al. (2009). (DOCX) [file pone.0194815.s004.docx]

**Supplementary Table 2**: Reference sequences used for phylogenetic analyses; includes strain codes, taxon name, host, country of collection, and GenBank accession numbers for the subset of isolates picked from Bischoff et al. (2009).

|  |  |  |  | **GenBank accession numbers** | |
| --- | --- | --- | --- | --- | --- |
| **Voucher #** | **Taxon** | **Isolation source** | **Country** | **EF-1α** | **Beta-tub** |
| 727 | *Metarhizium robertsii* | Orthoptera | Brazil | DQ463994 | EU248816 |
| 4739 | *Metarhizium robertsii* | Soil | Australia | EU248848 | EU248928 |
| 7501 | *Metarhizium robertsii* | Coleoptera | Australia | EU248849 | EU248929 |
| 4342 | *Metarhizium pingshaense* | Coleoptera | Solomon Islands | EU248851 | EU248821 |
| CBS 257.90 | *Metarhizium pingshaense* | Coleoptera | China | EU248850 | EU248820 |
| 3210 | *Metarhizium pingshaense* | Coleoptera | India | EQ463995 | EU248819 |
| 7929 | *Metarhizium pingshaense* | Isoptera | Australia | EU248847 | EU248815 |
| 7450 | *Metarhizium anisopliae* | Coleoptera | Australia | EU248852 | EU248823 |
| 7487 | *Metarhizium anisopliae* | Orthoptera | Eritrea | DQ463996 | EU248822 |
| 2107 | *Metarhizium brunneum* | Coleoptera | USA | EU248855 | EU248826 |
| 4179 | *Metarhizium brunneum* | Soil | Australia | EU248854 | EU248825 |
| 4152 | *Metarhizium brunneum* | Soil | Australia | EU248853 | EU248824 |
| 7505 | *Metarhizium majus* | Coleoptera | Australia | EU248870 | EU248842 |
| 4566 | *Metarhizium majus* | Coleoptera | Australia | EU248869 | EU248841 |
| 2808 | *Metarhizium majus* | Coleoptera | Philippines | EU248871 | EU248843 |
| 1914 | *Metarhizium majus* | Coleoptera | Philippines | EU248868 | EU248840 |
| 1946 | *Metarhizium majus* | Coleoptera | Philippines | EU248867 | EU248839 |
| 1015 | *Metarhizium majus* | Lepidoptera | Japan | EU248866 | EU248838 |
| 7502 | *Metarhizium guizhouense* | - | Australia | EU248861 | EU248833 |

**Supplementary Table 2 continued**

|  |  |  |  | **GenBank accession numbers** | |
| --- | --- | --- | --- | --- | --- |
| **Voucher #** | **Taxon** | **Isolation source** | **Country** | **EF-1α** | **Beta-tub** |
| 4321 | *Metarhizium guizhouense* | Soil | Australia | EU248860 | EU248832 |
| 7507 | *Metarhizium guizhouense* | Soil | Kiribati | EU248858 | EU248831 |
| CBS 258.90 | *Metarhizium guizhouense* | Lepidoptera | China | EU248862 | EU248834 |
| 6238 | *Metarhizium guizhouense* | Lepidoptera | China | EU248857 | EU248830 |
| 5714 | *Metarhizium guizhouense* | - | - | EU248856 | EU248829 |
| 7488 | *Metarhizium lepidiotae* | Coleoptera | Australia | EU248865 | EU248837 |
| 7412 | *Metarhizium lepidiotae* | Coleoptera | Australia | EU248864 | EU248836 |
| 4628 | *Metarhizium lepidiotae* | Soil | Australia | EU248863 | EU248835 |
| 7486 | *Metarhizium acridum* | Orthoptera | Niger | EU248845 | EU248813 |
